# Computational recognition and analysis of hitherto uncharacterized nucleotide cyclase-like proteins in bacteria

**DOI:** 10.1186/s13062-016-0130-9

**Published:** 2016-05-31

**Authors:** Gayatri Ramakrishnan, Abha Jain, Nagasuma Chandra, Narayanaswamy Srinivasan

**Affiliations:** Indian Institute of Science Mathematics Initiative, Indian Institute of Science, Bangalore, 560012 India; Undergraduate studies, Indian Institute of Science, Bangalore, 560012 India; Department of Biochemistry, Indian Institute of Science, Bangalore, 560012 India; Molecular Biophysics Unit, Indian Institute of Science, Bangalore, 560012 India

**Keywords:** Distant relationship, Domain of unknown function, Mycobacteria, Nucleotide cyclase, Protein evolution, Sequence analysis

## Abstract

**Abstract:**

Evolutionary relationship between class III nucleotide cyclases and an uncharacterized set of bacterial proteins from Actinobacteria, Bacteroidetes and Proteobacteria has been recognized and analyzed. Detailed analyses of sequence and structural features resulted in the recognition of potential cyclase function conferring residues and presence of signature topological motif (βααββαβ) in the uncharacterized set of bacterial proteins. Lack of transmembrane domains and signal peptide cleavage sites is suggestive of their cytosolic subcellular localization. Furthermore, analysis on evolutionarily conserved gene clusters of the predicted nucleotide cyclase-like proteins and their evolutionary relationship with nucleotide cyclases suggest their participation in cellular signalling events. Our analyses suggest expansion of class III nucleotide cyclases.

**Reviewers:**

This article was reviewed by Eugene Koonin and Michael Gromiha.

**Electronic supplementary material:**

The online version of this article (doi:10.1186/s13062-016-0130-9) contains supplementary material, which is available to authorized users.

## Background

The development of powerful homology recognition techniques over the past decade has aided in the establishment of protein structure-function and evolutionary relationships using sequence information alone. Sensitive search procedures that employ sequence-based and structure-based profiles have been shown to be effective in the reliable detection of remote evolutionary relationships, and are, therefore extensively used in protein structure and function prediction/recognition [1]. Such profile-driven search procedures have the potential of recognising interesting relationships not catalogued before. An in-house algorithm developed in the past, AlignHUSH, employs highly sensitive profile-profile alignment coupled with information on secondary structure probabilities and hydrophobicity of amino acids to recognise evolutionarily related protein families with reasonable accuracy [2]. This approach has been useful in deriving potential relationships between sequence and structural families, which is comprehended in our in-house database SUPFAM (http://supfam.mbu.iisc.ernet.in/) [3, 4]. The effectiveness of AlignHUSH is reflected in the recognition of relationship between a domain family of unknown function DUF2652 and nucleotide cyclase family, which forms the focus of this study.

Cyclic nucleotides are pivotal second messengers involved in intracellular signalling pathways in all organisms. The enzymes which synthesize cyclic nucleotides are nucleotidyl cyclases that catalyse the conversion of nucleotide triphosphates into their respective 3’,5’-cyclic nucleoside monophosphates (cNMP). Nucleotide cyclases are well-characterised family of enzymes which are categorised into six classes which differ in structure and catalytic mechanisms, suggesting independent evolution of these enzymes from diverse organisms. Of the six evolutionarily distinct classes, the family of class III nucleotidyl cyclases is the largest and the most diverse, spanning across eukaryotic and prokaryotic organisms [5]. The class III nucleotide cyclases comprise of a signature topological motif (βααββαβ) [6], function in a metal-dependent manner and are demonstrated to be catalytically active as dimers [5, 7].

Over the past decade, the identification of several prokaryotic nucleotide cyclases, especially bacterial cyclases, have revealed divergent protein domain architectures and variations in the catalytic domain [8]. In addition to this versatility, the bacterial nucleotide cyclases gain special interest due to the indispensability of cyclic nucleotides in the regulation of varied cellular processes, and in some pathogenic species, virulence pathways as well. A survey of nucleotide cyclases in Actinobacteria, demonstrated earlier [9], resulted in identification of sole occurrence of class III nucleotide cyclases in the bacterial phylum.

In the current study, we have presented analyses on probable structure and function acquired by a set of uncharacterised bacterial proteins on the basis of recognition of their evolutionary relationship with nucleotide cyclases. By means of comparative sequence and structural assessment, conservation of cyclase function conferring residues were recognised across all the bacterial proteins. Absence of transmembrane helices and signal peptide cleavage sites imply cytosolic subcellular localization of these proteins. Additionally, we have pursued analysis on gene neighbours of the proteins in question to identify probable functionally associated genes linked with signalling pathways. Owing to the well-studied diversity in bacterial class III nucleotide cyclases, our predictions could provide suitable grounds for exploration of an uncatalogued subfamily of nucleotide cyclases.

## Results and discussion

The primary grounds on which our analysis is based is the recognition of evolutionary relationship between the domain family of unknown function DUF2652 and nucleotide cyclase family determined using AlignHUSH at a reliable Z-score of 7.6 [10] (Additional file 1: Figure S1). To ascertain the relationship recognized, we assessed the overall fold acquired by the proteins of unknown structure and function pertaining to DUF2652 family using PHYRE^2^ [11] based on cues obtained using AlignHUSH. For all the proteins, the high confidence (>90 %) matches or the top scoring templates belonged to class III nucleotide cyclases. Indeed, the presence of topological motif (βααββαβ), preserved in nucleotide cyclases, was recognized. In addition, a recently updated release of InterPro database v.54 [12] was consulted, which suggested potential relationship between the uncharacterized protein family of interest and nucleotide cyclase family. However, the reliability of the relationship is not assessed so far and the function of uncharacterized protein family is yet to be identified. Therefore, we probed the structural and functional features shared between nucleotidyl cyclases and the proteins from DUF2652 family.

The uncharacterised non-redundant set of 53 bacterial proteins from DUF2652 family span 50 bacterial genomes with 22 pertaining to Actinobacteria (predominantly *Mycobacterium sp.*), 23 from Bacteroidetes and 8 from Proteobacteria lineage. With an average protein length of 250, these set of proteins were identified as single domain (DUF2652) containing proteins, except for one protein from *Bradyrhizobium* species [UniProt: U1IMSO] which constitutes N-terminal domain of unknown function DUF2652 and a polyketide cyclase domain at its C-terminal end.

### Nucleotide cyclase-like proteins in Mycobacteria

**Fig. 1:**
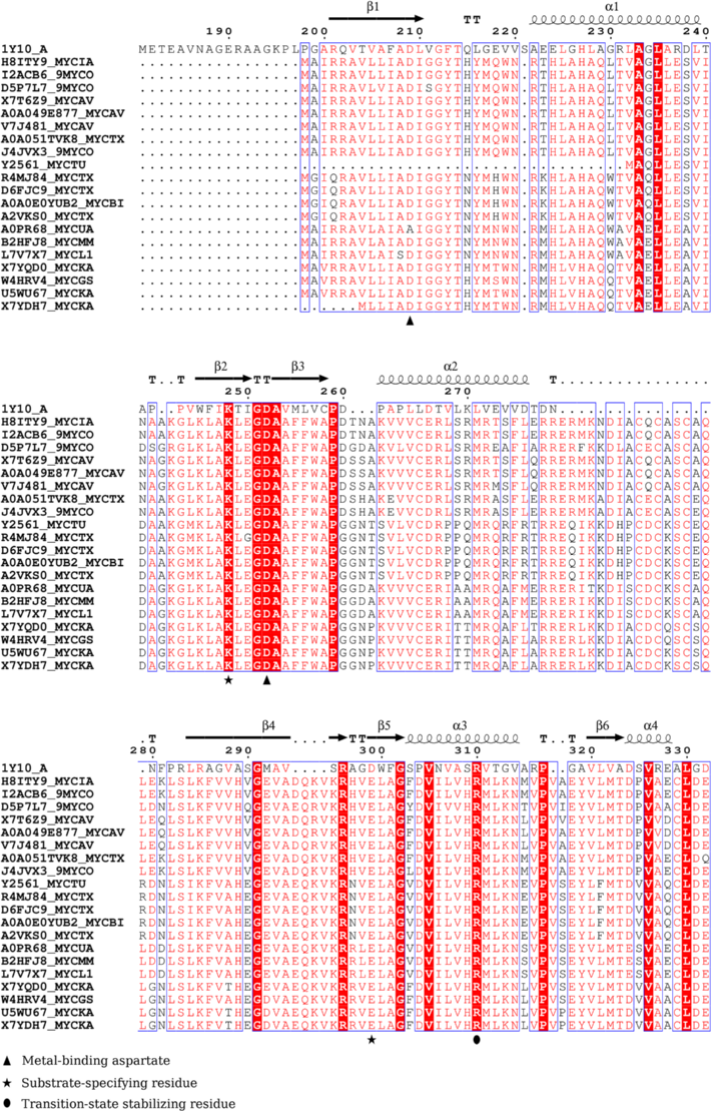
Inferences on functional residues in mycobacterial proteins. A multiple sequence alignment of 20 mycobacterial proteins of unknown structure and function with cyclase domain of Rv1264 was pursued (PDB code- 1Y10:A) using PROMALS3D. The alignment is rendered using ESPript (v.3.0) [34]. Only those aligned regions are shown which represent conservation of key functional residues. The secondary structural content and residue numbers have been extracted from Rv1264; α-helices are depicted as squiggles, β-strands as arrows and β-turns as tt letters. Columns coloured with red background denote strictly conserved residues (conservation score: 9, on a scale of 0–9 where nine denotes strict conservation); while the residues rendered as red coloured characters within blue frames depict reasonably well-conserved residues (conservation score: 6–8). The nucleotide cyclase function-conferring residues are tagged with symbols. This figure and Fig. 2 have been rendered in similar fashion

The catalytic activity of class III nucleotide cyclases is typically characterized by three features- 1) metal-binding residues, 2) substrate-binding residues, i.e., either ATP or GTP and 3) transition state-stabilizing residues. A set of 20 mycobacterial proteins of unknown structure and function were assessed for the presence of conservation of cyclase function-conferring residues. Figure 1 illustrates multiple sequence alignment of these proteins with cyclase domain of an established adenylyl cyclase Rv1264 from *M. tuberculosis*. In typical adenylyl cyclases, the catalytic residues are lysine and aspartate which establish preference for ATP by forming hydrogen bonds at positions N1 and N6 of adenine base of ATP [5]. Instead of Lys-Asp pair, Lys-Glu pair was found to be conserved across the 20 mycobacterial proteins (Fig. 1) which could potentially confer nucleotide substrate-specificity. The metal-binding aspartates indispensable for their role in catalytic activity of nucleotide cyclases were found to be conserved, except for Rv2561 [UniProt: Y2651] from *M. tuberculosis* H37Rv and MUL_2491 [UniProt: A0PR68] from *M. ulcerans*, which comprised only one of the conserved aspartates. Thus, the nucleotide cyclase function for these two homologues cannot be clearly attributed. Two transition state-stabilizing residues, asparagine and arginine in adenylyl cyclases, are believed to participate in the orientation of substrate and/or stabilizing the transition state intermediate for catalysis [5]. Of these two, only arginine was found to be conserved, while instead of asparagine, a conserved isoleucine was identified. This observation is similar to the atypical mycobacterial cyclase Rv1900c which comprises histidine in place of asparagine, thereby negating the requirement of asparagine for adenylyl cyclase activity. Due to the absence of asparagine equivalent, Rv1900c is purported to exhibit both adenylyl and guanylyl cyclase activity. Moreover, such an absence of asparagine equivalents have been observed in several established nucleotide cyclases [5]. Thus, on the basis of presence of glutamate in place of asparatate and absence of an asparagine equivalent in the set of the proteins assessed, it is likely that these nucleotide cyclase-like proteins of mycobacteria may confer specificity for both ATP and GTP.

### Guanylate cyclase-like proteins in Bacteroidetes

**Fig. 2:**
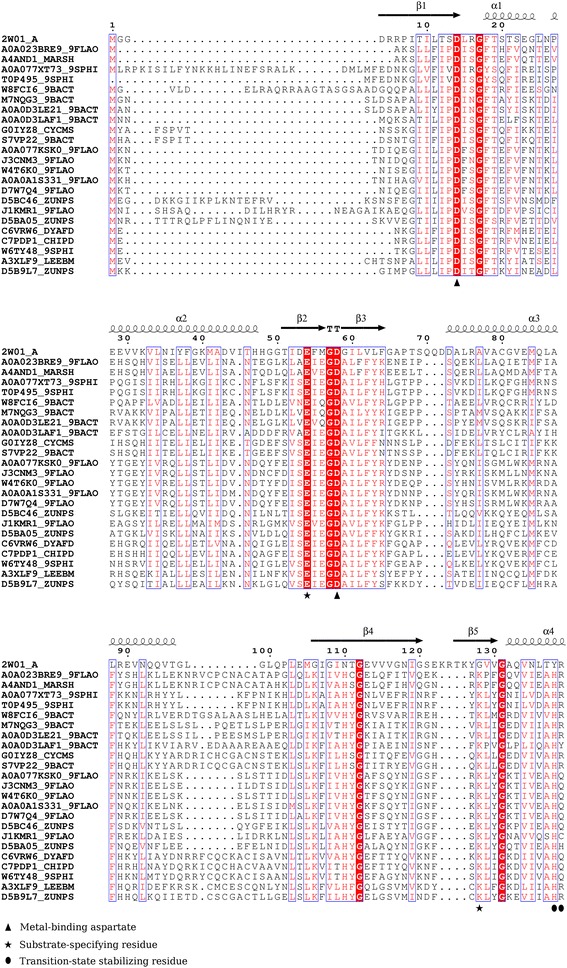
Inferences on functional residues in Bacteroidetes proteins. A multiple sequence alignment of 23 Bacteroidetes proteins with cyclase domain of cyanobacterial cyclase Cya2 (PDB code- 2W01:A) is depicted. . Only those aligned regions are shown which represent conservation of functional residues. The secondary structural content and residue numbers have been extracted from crystal structure of cyclase domain of Cya2

**Fig. 3:**
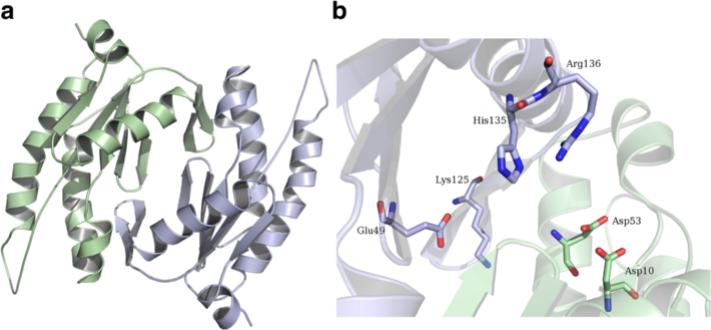
Elucidations on predicted guanylyl cyclase. **a** Modelled and energy-minimized homodimeric structure of [UniProt: A4AND1] from *Maribacter sp.*is illustrated; the two chains are shown in different colors. **b** Potential GTP-binding pocket of predicted guanylyl cyclase is illustrated with residues that are recognised to be conserved in Bacteroidetes proteins. The two aspartates are supplied by one of the subunits (*green*) while the rest of the residues are supplied by second subunit (*light blue*). This figure is generated using PyMOL (https://www.pymol.org) [35]

Similar to the analysis pursued for mycobacterial proteins, we assessed 23 proteins of Bacteroidetes lineage for the presence of residues which could potentially contribute to nucleotide cyclase activity. A multiple sequence alignment of these 23 proteins suggested presence of conserved Glu-Lys/Arg pair of residues at the position known to confer substrate-specificity in cyclases. To assess probable guanylyl cyclase function-conferring residues, multiple sequence alignment of 23 Bacteroidetes proteins with cyclase domain of cyanobacterial guanylyl cyclase Cya2 from *Synechocystis* sp. PCC6803 was undertaken (Fig. 2). Presence of glutamate at the position of lysine (as observed in adenylate cyclases) is usually indicative of guanylate cyclases and the preference for GTP-binding is conventionally known to be dictated by glutamate-cysteine pair. However, cysteine equivalent was found to be absent in the current set of 23 proteins, as is the case with cyanobacterial Cya2 as well. Cyanobacterial Cya2 comprises of unusual glutamate-glycine pair of residues, where glycine in place of cysteine is proposed to contribute in guanine base recognition by accommodating active site residues [13]. The active site lining residues in Cya2 include an unconventional tyrosine which is suggested to form hydrogen bond with guanine 2-amino group, and a typical glutamate residue which is suggested to interact with 2-amino group of guanine and NH group at position N1 of the base [13]. While the glutamate residue was identified to be conserved, in place of key residues i.e., glycine and tyrosine of Cya2 we recognised lysine/arginine and histidine, respectively, conserved across all 23 Bacteroidetes proteins (Fig. 2). Probable contributions of such conserved residues in interaction with GTP could be drawn. The presence of lysine/arginine at the position of glycine (indicated with asterisk in Fig. 2) may help in hydrogen bond formation with O6 of guanine, while histidine in place of tyrosine could possibly bind at position N7 of guanine base in GTP. Figure 3 exemplifies such a potential GTP-binding pocket in the modelled and energy-minimized homo-dimeric structure of one of the Bacteroidetes proteins [UniProt: A4AND1] from *Maribacter sp.* which was built based on crystal structure of cyanobacterial guanylyl cyclase Cya2.

The metal-binding aspartates were identified to be conserved across all the Bacteroidetes proteins in the current dataset. Arginine of cyanobacterial Cya2, proposed to participate in binding phosphate groups [13], was also found to be conserved across several proteins (Fig. 2). In addition, glutamine, histidine, tyrosine and cysteine residues at the same position in several other proteins, can be observed in the alignment, which have the potential to bind phosphate groups. With the inferences made, it is likely that the set of Bacteroidetes proteins assessed could be guanylate cyclase-like.

### Other proteins related to nucleotide cyclases

Eight uncharacterised proteins of Proteobacteria were assessed for the presence of nucleotide cyclase-like features. On the basis of nature of residues conserved as detailed in earlier subsections, we could determine 7 putative adenylyl cyclases and one potential guanylyl cyclase (Additional file 1: Figure S2a). Also, two proteins of Actinobacteria [UniProt: A0A024K0K6, UniProt: I4EWW4] from *Mycobacterium triplex* and *Modestobacter marinus,* respectively, could be recognised as a potential guanylyl cyclase (Additional file 1: Figure S2b).

It has been well-demonstrated earlier that the nucleotide cyclases are active only as dimers [5]. Typically in prokaryotes, the class III nucleotide cyclases function as homodimers, which is essentially catered by substrate-specificity conferring residues [14, 15] at the interface region. Based on the inferences drawn from (1) the structures thus acquired and (2) potential function determining residues in the bacterial proteins under study, it is plausible that these proteins are likely to function as homo-dimers.

We also examined the uncharacterised proteins for the presence of a highly conserved sequence motif Gly-Gly-Asp-Glu-Phe (GGDEF domain) which is typical of diguanylate cyclases in the family of class III nucleotide cyclases. The absence of this highly conserved sequence feature in all the proteins assessed and their poor structural similarity to GGDEF domain, indicate that these are unlikely to be diguanylate cyclases. Presence of highly conserved aspartates in almost all the bacterial proteins strongly suggests their participation in divalent metal cation coordination, which is critical for nucleotide cyclase activity. Furthermore, the lack of transmembrane helices and signal peptide cleavage sites in the proteins studied is suggestive of their cytosolic subcellular localization. The inferences thus made based on nature of conservation of residues across bacterial proteins imply a plausible unexplored subfamily of soluble class III adenylyl or guanylyl cyclases. Table 1 summarises the details for 53 potential nucleotide cyclases.

**Table 1:** Details on 53 uncharacterised bacterial proteins related to nucleotide cyclases. The description of these proteins is provided in Additional file 3a

Sr. No.	UniProt ID	Protein length	Bacterial lineage	Key residues^a^			Predicted nucleotide cyclase
				MB	SS	TS	
1.	A0A023BRE9	362	Bacteroidetes	D, D	E, K	H, R	GC
2.	A0A024K0K6	242	Actinobacteria	D, D	E, K	H, R	GC
3.	A0A049E877	242	Actinobacteria	D, D	K, E	R	AC/GC
4.	A0A051TVK8	242	Actinobacteria	D, D	K, E	R	AC/GC
5.	A0A077KSK0	184	Bacteroidetes	D, D	E, K	H, Q	GC
6.	A0A077XT73	217	Bacteroidetes	D, D	E, K	H, R	GC
7.	A0A0A1S331	184	Bacteroidetes	D, D	E, K	H, Q	GC
8.	A0A0D3LAF1	210	Bacteroidetes	D, D	E, K	H, Y	GC
9.	A0A0D3LE21	204	Bacteroidetes	D, D	E, R	H, R	GC
10.	A0A0E0YUB2	245	Actinobacteria	D, D	K, E	R	AC/GC
*11.*	*A0PR68*	*213*	*Actinobacteria*	*D*	*K, E*	*R*	*AC/GC*
12.	A2VKS0	245	Actinobacteria	D, D	K, E	R	AC/GC
13.	A3XLF9	209	Bacteroidetes	D, D	E, K	H, R	GC
14.	A4AND1	360	Bacteroidetes	D, D	E, K	H, R	GC
15.	B2HFJ8	244	Actinobacteria	D, D	K, E	R	AC/GC
16.	C6VRW6	359	Bacteroidetes	D, D	E, K	H, Q	GC
17.	C7PDP1	360	Bacteroidetes	D, D	E, K	H, Q	GC
18.	D5B9L7	192	Bacteroidetes	D, D	E, K	H, R	GC
19.	D5BA05	215	Bacteroidetes	D, D	E, K	H, H	GC
20.	D5BC46	217	Bacteroidetes	D, D	E, K	H, Q	GC
21.	D5P7L7	242	Actinobacteria	D, D	K, E	R	AC/GC
22.	D6FJC9	245	Actinobacteria	D, D	K, E	R	AC/GC
23.	D7W7Q4	183	Bacteroidetes	D, D	E, K	H, Q	GC
24.	E3FM84	240	Proteobacteria	D, D	K, E	R	AC/GC
25.	F8C8H4	241	Proteobacteria	D, D	K, E	R	AC/GC
26.	G0IYZ8	370	Bacteroidetes	D, D	E, K	H, R	GC
27.	H8ITY9	242	Actinobacteria	D, D	K, E	R	AC/GC
28.	H8MLI0	243	Proteobacteria	D, D	K, E	R	AC/GC
29.	I2ACB6	242	Actinobacteria	D, D	K, E	R	AC/GC
30.	I4EWW4	239	Actinobacteria	D, D	E, K	H, R	GC
31.	J1KMR1	196	Bacteroidetes	D, D	E, K	H, C	GC
32.	J3CNM3	188	Bacteroidetes	D, D	E, K	H, Q	GC
33.	J4JVX3	242	Actinobacteria	D, D	K, E	R	AC/GC
34.	L7U9J7	249	Proteobacteria	D, D	K, E	R	AC/GC
35.	L7V7X7	244	Actinobacteria	D, D	K, E	R	AC/GC
36.	M7NQG3	205	Bacteroidetes	D, D	E, R	H, R	GC
*37.*	*P9WL99*	*212*	*Actinobacteria*	*D*	*K, E*	*R*	*AC/GC*
38	Q1DBS3	241	Proteobacteria	D, D	K, E	R	AC/GC
39.	R4MJ84	245	Actinobacteria	D, D	K, E	R	AC/GC
40.	S7VP22	364	Bacteroidetes	D, D	E, K	H, R	GC
41.	T0P495	189	Bacteroidetes	D, D	E, K	H, R	GC
42.	U1IMS0	385	Proteobacteria	D, D	K, E	R	AC/GC
43.	U2U286	241	Proteobacteria	D, D	K, E	R	AC/GC
44.	U5WU67	242	Actinobacteria	D, D	K, E	R	AC/GC
45.	V7J481	242	Actinobacteria	D, D	K, E	R	AC/GC
46.	W4HRV4	242	Actinobacteria	D, D	K, E	R	AC/GC
47.	W4M0R9	361	Proteobacteria	D, D	E, K	H, R	GC
48.	W4T6K0	183	Bacteroidetes	D, D	E, K	H, Q	GC
49.	W6TY48	354	Bacteroidetes	D, D	E, K	H, Q	GC
50.	W8FCI6	383	Bacteroidetes	D, D	E, K	H, R	GC
51.	X7T6Z9	242	Actinobacteria	D, D	K, E	R	AC/GC
52.	X7YDH7	236	Actinobacteria	D, D	K, E	R	AC/GC
53.	X7YQD0	242	Actinobacteria	D, D	K, E	R	AC/GC

^a^The key conserved residues are categorised into metal-binding (MB), substrate-specifying (SS) and transition-state (TS) or other stabilising residues. Single-letter codes for amino acids are used. Entries in italics highlight the proteins with single metal-binding aspartate

### Analysis on evolutionary divergence

**Fig. 4:**
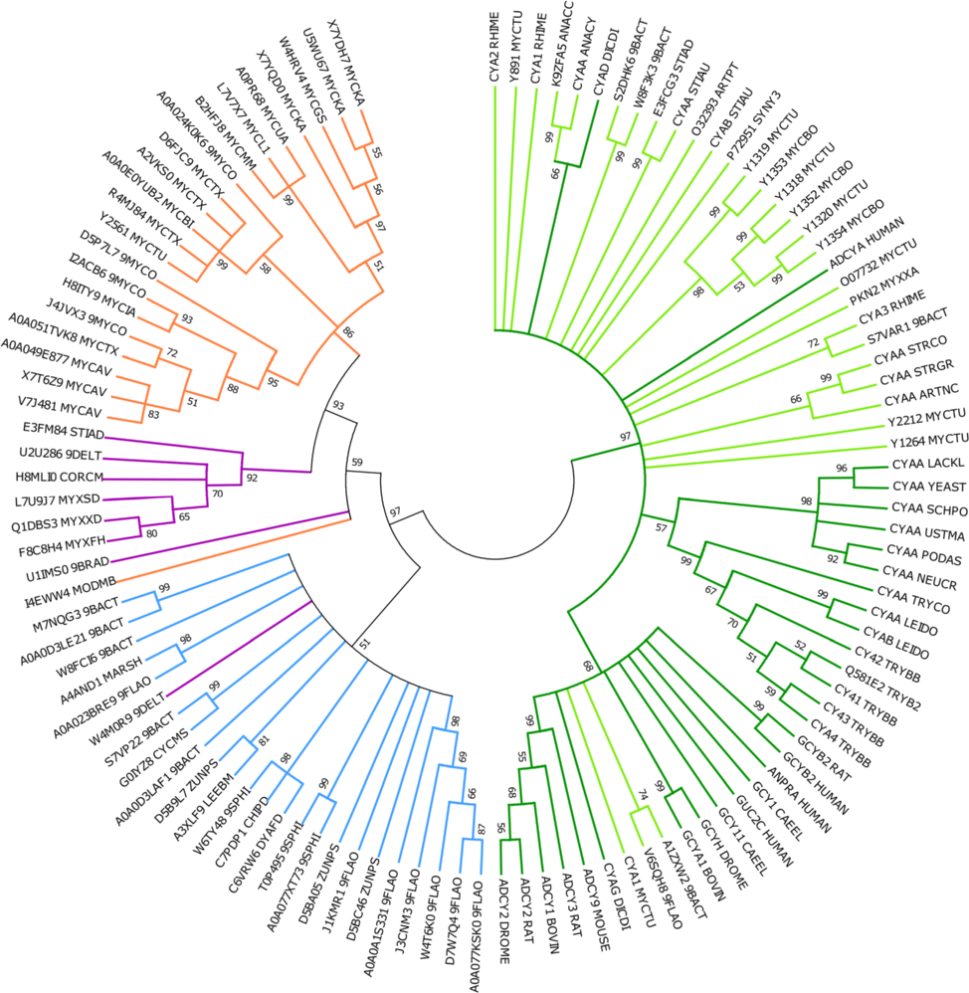
Evolutionary divergence. A neighbour-joining tree is shown which was bootstrapped at a cut-off of 50 %. These values are mentioned on the nodes in the circular un-rooted tree. The coloured branches depict established cyclases from eukaryotes (*dark green*), established cyclases from prokaryotes (*light green*) and nucleotide cyclase-like proteins from Actinobacteria (*orange*), Bacteroidetes (*blue*) and Proteobacteria (*purple*). The analysis involved 113 proteins and was conducted using MEGA 6. Details on gene IDs of established cyclases is provided in the supplementary material (Additional file 2)

The low sequence identity of the bacterial nucleotide cyclase-like proteins under study with established cyclases (<25 %), albeit constituting key structural and functional features, prompted further analysis on estimating extent of evolutionary divergence. The cyclase domains of experimentally verified nucleotide cyclases from several eukaryotes as well as from three bacterial phyla, i.e., Actinobacteria, Bacteroidetes and Proteobacteria (see Additional file 2), together with the set of bacterial proteins under investigation were analysed by means of sequence similarity-based clustering. As illustrated in Fig. 4, two distinct clusters are readily apparent. It is interesting to note that all the established cyclases from the three bacterial phyla cluster with eukaryotic nucleotide cyclases. The disparate nature of the two clusters, i.e., established and predicted cyclases, is suggestive of a probable evolutionarily distinct subfamily of class III nucleotide cyclases. Also evident from the figure is the clustering of Proteobacteria proteins (purple) with the proteins from Actinobacteria (orange), mainly Mycobacteria. This finding can be justified based on details on the conserved sequence features indicative of nucleotide cyclase-like proteins in Mycobacteria and Proteobacteria capable of conferring specificity for both ATP and GTP. Likewise, one protein from Proteobacteria [UniProt: W4M0R9] is observed to cluster with guanylate cyclase-like proteins of Bacteroidetes.

### Probable functional associations of predicted nucleotide cyclases

Functionally coupled genes are often closely organized in prokaryotic genomes. Such gene clusters, when conserved across multiple genomes, usually imply participation of the encoded proteins in a biological pathway [16]. Also, functional interactions between proteins can be predicted by Rosetta stone method, which suggests the likelihood of interaction between single-domain containing proteins encoded by separate genes in a genome, that occur as multi-domain proteins due to gene fusion, either in the same or other organisms [17]. Based on these well-demonstrated concepts, we made an attempt to recognise interactions between nucleotide cyclase-like and their gene neighbours, which can further support our predictions made on their involvement in signalling pathways.

The nucleotide cyclase domain family comprises of 746 domain architectures as per the information provided in Pfam database [18]. We pursued a comparative assessment of protein domain architectures of gene neighbours of all 84 proteins (53 proteins along with redundant instances) and those of cyclase domain containing proteins. The domain architectures of gene products of only those gene neighbours were considered which are conserved in closely related genomes, i.e., evolutionarily conserved gene clusters. We identified 20 probable functional associations for 12 predicted nucleotide cyclases in our dataset on the basis of 5 distinct domains coexisting with cyclase domains, as observed in multi-domain nucleotide cyclases. The domain architectures were mainly constituted by two domains- a cyclase domain succeeding or preceding one of the following: alpha/beta hydrolase domain, cyclic nucleotide-binding domain, helix-turn-helix motif, phosphohydrolase domain or periplasmic binding domain (Additional file 1: Figure S3). The list of probable functionally associated genes is detailed in Table 2. Interestingly, several predicted associations were with those domains (in the partner proteins) that are typically involved in signal transduction pathways and regulatory processes such as cNMP-binding domain, thus supporting the predictions made on the involvement of the uncharacterized set of bacterial proteins in cellular signalling events.

**Table 2:** Probable functional associations recognised for potential nucleotide cyclases

Sr. No.	UniProt ID	Gene name	Potential interacting partner	Pfam domain of the interacting partner [Pfam ID]	Gross function of the interacting partner
1.	R4MJ84	J113_17860	J113_17870	Cyclic nucleotide-binding domain [PF00027]	Signal transduction, regulatory processes
2.	R4MJ84	J113_17860	J113_17875	Cyclic nucleotide-binding domain [PF00027]	Signal transduction, regulatory processes
3.	J9WHW1	MIP_02960	MIP_02970	Periplasmic binding protein [PF13458]	Signal transduction, transport
4.	J9WHW1	MIP_02960	MIP_02959	Alpha/beta hydrolase domain [PF12697]	Metabolic processes
5.	U5WU67	MKAN_22035	MKAN_22050	Alpha/beta hydrolase domain [PF12697]	Metabolic processes
6.	B2HFJ8	MMAR_1384	MMAR_1388	Alpha/beta hydrolase domain [PF12697]	Metabolic processes
7.	B2HFJ8	MMAR_1384	MMAR_1378	Helix-turn-helix domain [PF01381]	Regulatory processes
8.	P9WL98	MT2638	MT2640	Cyclic nucleotide-binding domain [PF00027]	Signal transduction, regulatory processes
9.	P9WL98	MT2638	MT2641	Cyclic nucleotide-binding domain [PF00027]	Signal transduction, regulatory processes
10.	A0PR68	MUL_2491	MUL_2488	Alpha/beta hydrolase domain [PF12697]	Metabolic processes
11.	A0PR68	MUL_2491	MUL_2497	Helix-turn-helix domain [PF01381]	Regulatory processes
12.	L7V7X7	MULP_01555	MULP_01559	Alpha/beta hydrolase domain [PF12697]	Metabolic processes
13.	L7V7X7	MULP_01555	MULP_01549	Helix-turn-helix domain [PF01381]	Regulatory processes
14.	H8ITY9	OCU_21180	OCU_21250	Periplasmic binding protein [PF13458]	Signal transduction, transport
15.	H8ITY9	OCU_21180	OCU_21170	Alpha/beta hydrolase domain [PF12697]	Metabolic processes
16.	S4Z758	OEM_18810	OEM_18800	Alpha/beta hydrolase domain [PF12697]	Metabolic processes
17.	P9WL99	Rv2561	Rv2564	Cyclic nucleotide-binding domain [PF00027]	Signal transduction, regulatory processes
18.	P9WL99	Rv2561	Rv2565	Cyclic nucleotide-binding domain [PF00027]	Signal transduction, regulatory processes
19.	I2ACB6	W7S_09825	W7S_09820	Alpha/beta hydrolase domain [PF12697]	Metabolic processes
20	W8FCI6	HSW_3800	HSW_3799	Phosphohydrolase [PF01966]	Nucleic acid metabolism, signal transduction

## Conclusions

Exploration of evolutionary relationships with sensitive profile-based search procedures has the potential of recognizing putative sequence-function and structure-function relationships not documented earlier. With the discovery of unconventional class III nucleotide cyclases, the repertoire of nucleotidyl cyclase-like proteins is broadened. Using a survey of about 50 bacterial genomes, we have described details on structural and functional features of hitherto uncharacterized proteins related to class III nucleotide cyclases. The 3-D fold of these proteins as recognized by fold recognition methods indicating clear evolutionary relationship with nucleotidyl cyclases, together with presence of conserved metal-binding aspartates, conservation of substrate-specifying residues, presence of transition-state stabilizing residues and sequence similarity-based clustering analysis, imply the possibility of an unexplored evolutionarily distinct subfamily of class III nucleotide cyclases. Analysis on evolutionarily conserved gene clusters of the nucleotide cyclase-like proteins revealed functional associations that support the predictions on their participation in cellular signalling events. The inferences presented in this study are suggestive of cyclase-like structure and function of the uncharacterized bacterial proteins, thus rendering a scope for potential expansion of class III nucleotidyl cyclases. In-depth experimental investigations of these proteins can further concretize evidence on their possible cellular roles and in addition, can aid in understanding the pathobiology of pathogenic species of interest.

## Methods

### Data set: DUF2652 family

According to the Pfam database (Pfam v.28), DUF2652 is a family of proteins of no known structure and function. This family, with a profile length of 118 residues, comprises of 85 proteins, of which 84 belong to three bacterial lineages: Actinobacteria, Bacteroidetes and Proteobacteria, and the remaining one member is a protein predicted from marine sediment metagenome DNA, contig: S01H1_L10677. For in-depth analysis this protein was excluded from the study as the source organism is unknown or belongs to an unclassified lineage. Thus, protein sequence information for 84 proteins containing DUF2652 domain was obtained from Pfam database. A recently updated release of Pfam database (v.29) was also consulted. The initial set of proteins from DUF2652 family retrieved from Pfam database contained instances which were recognized as incomplete or partial gene products based on gene records in NCBI RefSeq [19] and Ensembl [20] databases. In four cases, one complete gene was annotated as two distinct adjacently localized genes, believed to encode for two gene products. For instance, Rv2561 and Rv2562 of *M. tuberculosis* H37Rv are annotated as two distinct genes in TubercuList database [21]. The updated gene records in UniProt database [22] and NCBI RefSeq have produced a revised version where Rv2561 and Rv2562 are merged as one gene record [UniProt: P9WL99]. Similar “split” cases were recognized, however, information on gene completeness was not available. Thus, the protein sequences pertaining to such genes were not considered in the analysis as the gene products are questionable regarding their completeness. Additional file 1: Table S1 details such entries that have not been taken into account.

### Sequence analysis

Profile-HMM models and protein sequences of unknown structure and function pertaining to the domain family DUF2652 were obtained from Pfam v.28. The sequence dataset was augmented using jackhmmer, available from HMMER3.1 suite of programs [23], against non-redundant database of over 60 million sequences (obtained from NCBI database) at an E-value threshold of 0.0001, sequence identity cut-off of 25 %, 10 iterations and an alignment coverage cut-off of 60 %. All the hits identified corresponded to bacterial proteins of unknown structure and function. The resultant hits along with the set of proteins retrieved from Pfam were clustered at 100 % sequence identity using blastclust (ftp://ftp.ncbi.nih.gov/blast/documents/blastclust.html), to achieve a final set of 53 non-redundant representative set of bacterial proteins of unknown structure and function. The details on the proteins excluded from the analysis is provided in Additional file 3b. Of the 53 non-redundant proteins, 21 were recognised as proteins from *Mycobacterium* genus and 32 were non-mycobacterial which included organisms from other bacterial (Proteobacteria and Bacteroidetes) lineages.

Multiple sequence alignments of 53 uncharacterised bacterial proteins were obtained using PROMALS3D (PROfile Multiple Alignment with predicted Local Structures and three-dimensional constraints) [24] along with protein sequences of established nucleotidyl cyclases based on cues obtained from AlignHUSH, as explained earlier. The conservation of nature and position of key cyclase function conferring residues in nucleotide cyclases were considered in the aligned stretch of residues to infer the probable function of uncharacterised set of bacterial proteins.

In order to estimate the extent of evolutionary divergence, protein sequences of established nucleotide cyclases from human, rat, yeast and several other eukaryotic genomes as well as those of established cyclases from Actinobacteria, Bacteroidetes and Proteobacteria were retrieved from UniProt database and clustered at 60 % sequence identity using blastclust to arrive at a feasible representative set of established cyclases. A multiple sequence alignment of cyclase domains of representative eukaryotic and bacterial organisms together with 53 bacterial proteins was achieved using MUSCLE [25]. This alignment was further subjected to evolutionary analysis pursued using Molecular Evolutionary Genetics Analysis (MEGA) tool version 6 [26]. Presence of potential transmembrane helices and signal peptide cleavage sites in the uncharacterised bacterial proteins were checked using TMHMM (version 2.0) [27] and SignalP (version 4.1) [28].

### Analysis of 3-D structure

The probable fold acquired by the bacterial proteins of unknown structure and function was identified using the fold recognition program PHYRE^2^. PHYRE^2^ associates a fold with a confidence measure, which if >90 %, implies correctness of the recognised fold for a query protein sequence according to a benchmark study [29]. For a selected case in the current analysis, structural model was generated using MODELLER (version 9.15) [30] and assessed for reliability using z-DOPE score (Discrete Optimized Protein Energy) and a query coverage threshold of 60 %. The modelled structure was energy-minimised using CHARMM force field calculations availed from GROMACS toolkit (version 4.5.5) [31].

### Analysis on genomic context of uncharacterised proteins and their domain architectures

The gene neighbours of all the uncharacterised proteins were obtained from NCBI database and the information on conserved gene neighbourhood was retrieved from SSDB (Sequence Similarity Database) availed through KEGG resource [32]. Protein sequences and annotations of gene products of the gene neighbours were obtained from UniProt database [22]. This exercise facilitated identification of potential functionally “coupled” gene clusters. To identify functionally coupled gene clusters associated with signalling pathways, Rosetta stone approach [17] was used, which suggests that two proteins/domains, for instance A and B, have a better-than-random chance of interacting when their homologues in another organism exist as a fused protein A-B. Thus, comparative analysis on domain architectures observed in established nucleotide cyclases and those observed in the gene neighbours of uncharacterized proteins was pursued. For domain assignment, these proteins were subjected to hmmscan, a program available through HMMER3.1 package. The profile-HMMs used were of Pfam domain families (16,230) which constitute information on domain family-specific gathering thresholds, manually curated by curators of Pfam database. To extract reliable domain assignments we used these thresholds as they correspond to an E-value cut-off of 0.01 [33]. Information on domain architectures for the uncharacterised proteins, gene products of their gene neighbours and the members of nucleotide cyclase domain family was obtained from Pfam. A schematic representation of the steps taken to identify functionally coupled gene clusters associated with signalling pathways is illustrated in Additional file 1: Figure S4.

## Abbreviations

AC, adenylyl cyclase; AlignHUSH, alignment of HMMs using structural and hydrophobicity information; ATP, adenosine triphosphate; cAMP, 3’-5’-cyclic adenosine monophosphate; c-di-GMP, cyclic di-guanosine monophosphate; cGMP, 3’-5’-cyclic guanosine monophosphate; CHARMM, chemistry at Harvard molecular mechanics; cNMP, cyclic nucleoside monophosphate; DUF, domains of unknown function; GC, guanylyl cyclase; GROMACS, Groningen machine for chemical simulations; GTP, guanosine triphosphate; HMM, hidden Markov model; KEGG, kyoto encyclopaedia of genes and genomes; MEGA, molecular evolutionary genetics analysis; NCBI, national center for biotechnology information; Pfam, protein family database; PHYRE^2^, protein homology recognition engine; PROMALS3D, profile multiple alignment with predicted local structures and three-dimensional constraints; SSDB, sequence similarity database

## Reviewer’s comments

### Reviewer’s report 1: Eugene Koonin

Reviewer comments: Ramakrishnan and colleagues predict a new family of bacteria class III nucleotide cyclases. The prediction is quite robust, I have easily verified it. They also analyze the genomic context of the DUF2652 which is compatible with cyclase activity even if not particularly important. Experimental validation of this prediction will be of interest, particularly, perhaps, because such a protein is encoded by Mycobacterium tuberculosis and other Mycobacteria.

The manuscript is much too long for a Discovery Note and not structured appropriately. It is my strong opinion that there is no need to expand this to a full research paper. The results are fully commensurate with the Discovery Note format, so I believe that the manuscript has to be shortened substantially, with most of the figures either dropped or moved to the supplement. Furthermore, the reported findings are more straightforward than a reader of the paper would be impressed to think. Indeed, the significant similarity of the DUF2652 proteins to adenylate cyclases is readily detected by the 2nd iteration of PSI-BLAST as well as HHPred. This is not to trivialize the finding which is indeed beyond the standard annotation pipelines. However, it is necessary to indicate that simple tools that are routinely used to perform more sensitive sequences analysis easily detect the relationship, so although the authors are certainly entitled to use their own software, it is not strictly necessary.

Response: *We thank Dr. Koonin for the useful comments and valuable suggestions. As suggested, we have shortened the manuscript substantially. We have moved four figures to the supplement and shortened the text content. In our study, the reliable detection of evolutionary relationship across proteins of DUF2652 family and nucleotide cyclases, using AlignHUSH, prompted us to investigate the relationship further. Indeed, standard tools such as PSI-BLAST also identify the said relationship, however, with questionable significance (depending upon the size of sequence database). Since the nucleotide cyclases and the uncharacterized set of bacterial proteins share less than 25 % sequence identity, it becomes necessary to investigate the reliability of the relationship using diverse methods. Studies on nucleotide cyclases in bacteria gain special interest due to their diverse nature and indispensability of cyclic nucleotides in various cellular roles.*

### Reviewer’s report 2: Michael Gromiha

Reviewer comments: In this work, the authors analyzed the sequence and structural features of class III nucleotide cyclases and an uncharacterized set of bacterial proteins and identified a motif in the uncharacterized proteins. Further, they analyzed the conservation of gene clusters and localization of such proteins to understand their participation in cell signaling events. The work is interesting and it provides insights about uncharacterized proteins. The manuscript could be improved with the following suggestions.

Comment: 1. Initially 83 proteins were considered and only 53 were used in the final set by comparing with sequence identity. The details about other 31 proteins could be given.

Response: *We thank Professor Gromiha for his comments and suggestions.*

*The details on the proteins (including incomplete gene records), excluded from the main analysis, are provided in Additional file**3**b.*

Comment: 2. It is mentioned that the alignment coverage of 60 % was used. It is not clear about the sequence identity.

Response: *The percentage sequence identity cut-off used for the generation of augmented sequence dataset was 25 %. This point is now included in page 13 in the revised version of the manuscript.*

Comment: 3. Later it has been mentioned that blastclust was used with a sequence identity of 60 %. The cutoff may be justified.

Response: *The protein sequences of established nucleotide cyclases were clustered at a sequence identity cut-off of 60 % to arrive at a representative set of proteins that can be further analysed for their relationship with the bacterial proteins under study. This point is now mentioned in page 14 in the revised version of the manuscript.*

*Proteins with very high sequence identity are likely to be clustered quite closely and the results of search for homologues with such closely-related proteins as query are likely to be highly similar. Therefore, we chose a cut-off value of 60 % in blastclust and this cut-off is reasonable to avoid inclusion of very closely related proteins in searches and phylogenetic analyses.*

Comment: 4. It is better to provide the details about functional annotation, structural classes/folds etc. for the set of proteins used in the study in Table 1.

Response: *The set of proteins used in the study are members of DUF2652 family which are uncharacterized proteins of no known structure and function. Thus, the proteins listed in Table*1*do not have any information on function, structural class or fold in publicly available databases. Based on our study, we predict with reasonable confidence that the set of uncharacterized bacterial proteins are potential class III nucleotide cyclases. We have included a supplementary table (Additional file**3**a) detailing 53 proteins as described in UniProt, in the revised version of manuscript.*

Comment: 5. It is assumed that the authors used conservation if all residues are the same in a sequence position. It could be mentioned clearly. Otherwise, the program used to compute the score and the score may be given.

Response: *We have used both nature and position of key cyclase function conferring residues in nucleotide cyclases to infer potential cyclase activity of the uncharacterised bacterial proteins. We have added this statement in page 13 in the revised version of the manuscript. We have also added information on conservation scores of residues in the aligned regions in Figure 1 and in the figure legend of the revised manuscript.*

Comment: 6. The conservation details of key residue shown in Figure 1 may be included in Figures 2 and 3.

Response: *The conservation details of key residues shown in Figure 1 are also shown in detail in Figures 2, 3 and 5. The key to the symbols used are described within each of the figures. Figures 1 and 5 have been moved to Additional file**1**in the revised version of the manuscript.*

Comment: 7. The significance of conserved residues shown in Figures 2 and 3, which are not in a part of metal binding, substrate binding and transition state stabilizing residues may be discussed.

Response: *The highly conserved residues not known to participate in cyclase activity are majorly non-polar residues such as alanine, valine and leucine or residues predominantly found in turns such as glycine and proline. It is likely that such residues contribute to the structural stability of the protein.*

Comment: 8. It is recommended to proofread for English corrections. For example, page 4, lines 82–83, the sentence, “Absence of …” is not clear; Page 2, Findings may be replaced with “Background”. Page 5, line 98, “cues”; page 13, line 394, The updated, space is missing etc.

Response: *The manuscript has been revised and corrected based on the suggestions.*

## Additional files

Additional file 1: Supplementary figures and a table. (DOCX 1515 kb) Additional file 2: Detailed list of gene IDs of established nucleotide cyclases from eukaryotes and bacteria used in Fig. 4. (XLSX 14 kb) Additional file 3: A) Description of proteins used in the study. B) Description of proteins excluded from the study. (XLSX 19 kb)
