# Supplementary material for: Computational recognition and analysis of hitherto uncharacterized nucleotide cyclase-like proteins in bacteria
Source: Biol Direct. 2016 May 31;11:27. doi: 10.1186/s13062-016-0130-9 (PMC4886424; doi:10.1186/s13062-016-0130-9)
Supplement: Additional file 1: — Supplementary figures and a table. (DOCX 1515 kb) [file 13062_2016_130_MOESM1_ESM.docx]

**Additional file 1**

**Supplementary Figures**


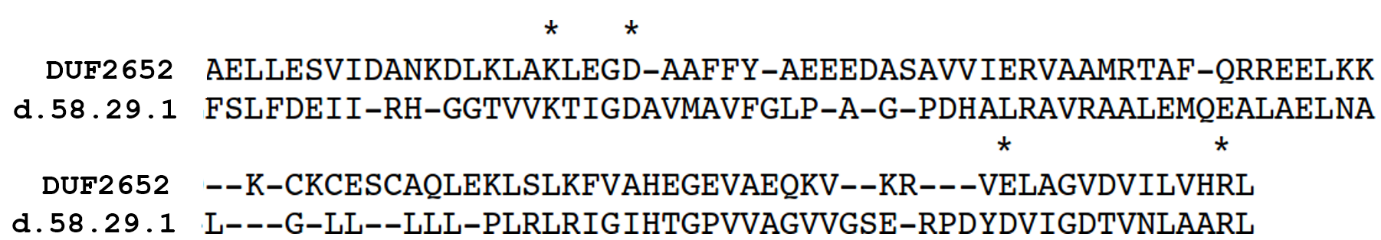


Figure-S1: The HMM-HMM alignment of DUF2652 (region: 38-116) and SCOP family d.58.29.1 (region: 152-199) at a Z-score of 7.6, as obtained from AlignHUSH is shown. The SCOP code d.58.29.1 corresponds to proteins comprising adenylyl and guanylyl cyclase catalytic domain. The critical residues for nucleotidyl cyclase activity, also conserved in the DUF family, are highlighted with asterisks.


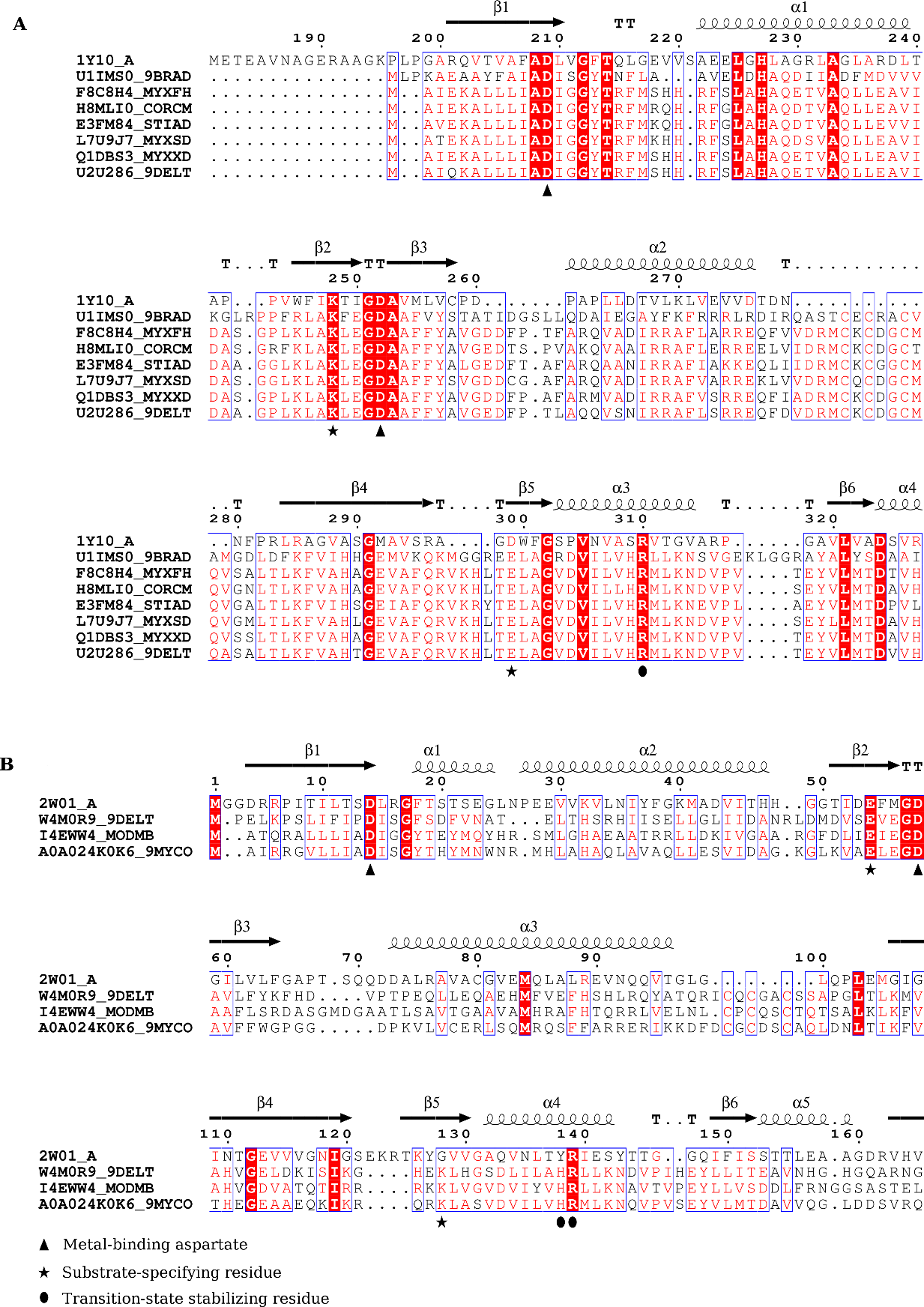


Figure-S2: Inferences on functional residues in other bacterial proteins. A) Multiple sequence alignment of 7 Proteobacteria proteins with cyclase domain of Rv1264 (PDB code- 1Y10:A) is depicted. B) Multiple sequence alignment of three proteins, two from Actinobacteria [UniProt: A0A024K0K6, UniProt: I4EWW4] from *Mycobacterium triplex* and *Modestobacter marinus,* respectively, and one from Proteobacteria [UniProt: W4M0R9] with cyclase domain of established guanylyl cyclase Cya2 is shown. Only those aligned regions are shown which represent conservation of functional residues.


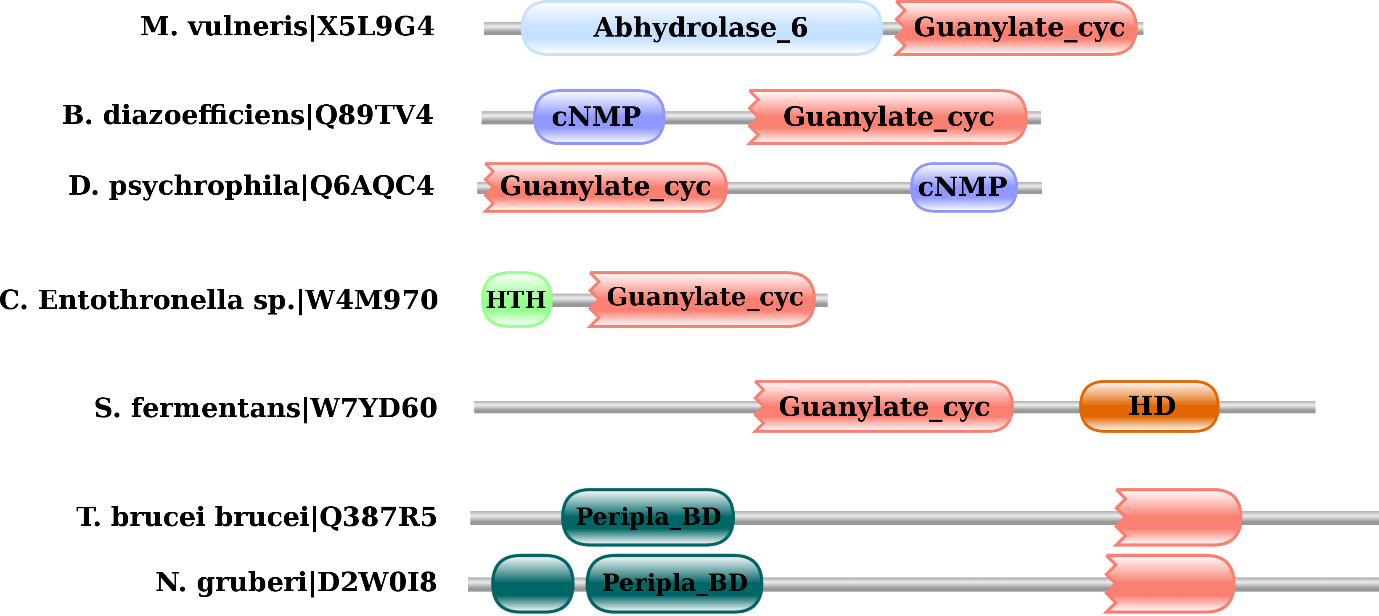


Figure-S3: Domain architectures of established nucleotide cyclases. Schematic representations (Pfam-style domain graphics) of protein domain architectures are drawn for those nucleotide cyclases that comprise of a domain other than cyclase, which is also identified in the gene neighbours of nucleotide cyclase-like proteins. The length of the domains and their proximities, shown in the figure, are equivalent to their approximate positions in a protein sequence. The un-labelled domains correspond to the Pfam domains- Guanylate_cyc (peach) and Peripla_BD (dark green). Note: the Pfam domain Guanylate_cyc comprises of adenylate and guanylate cyclases.


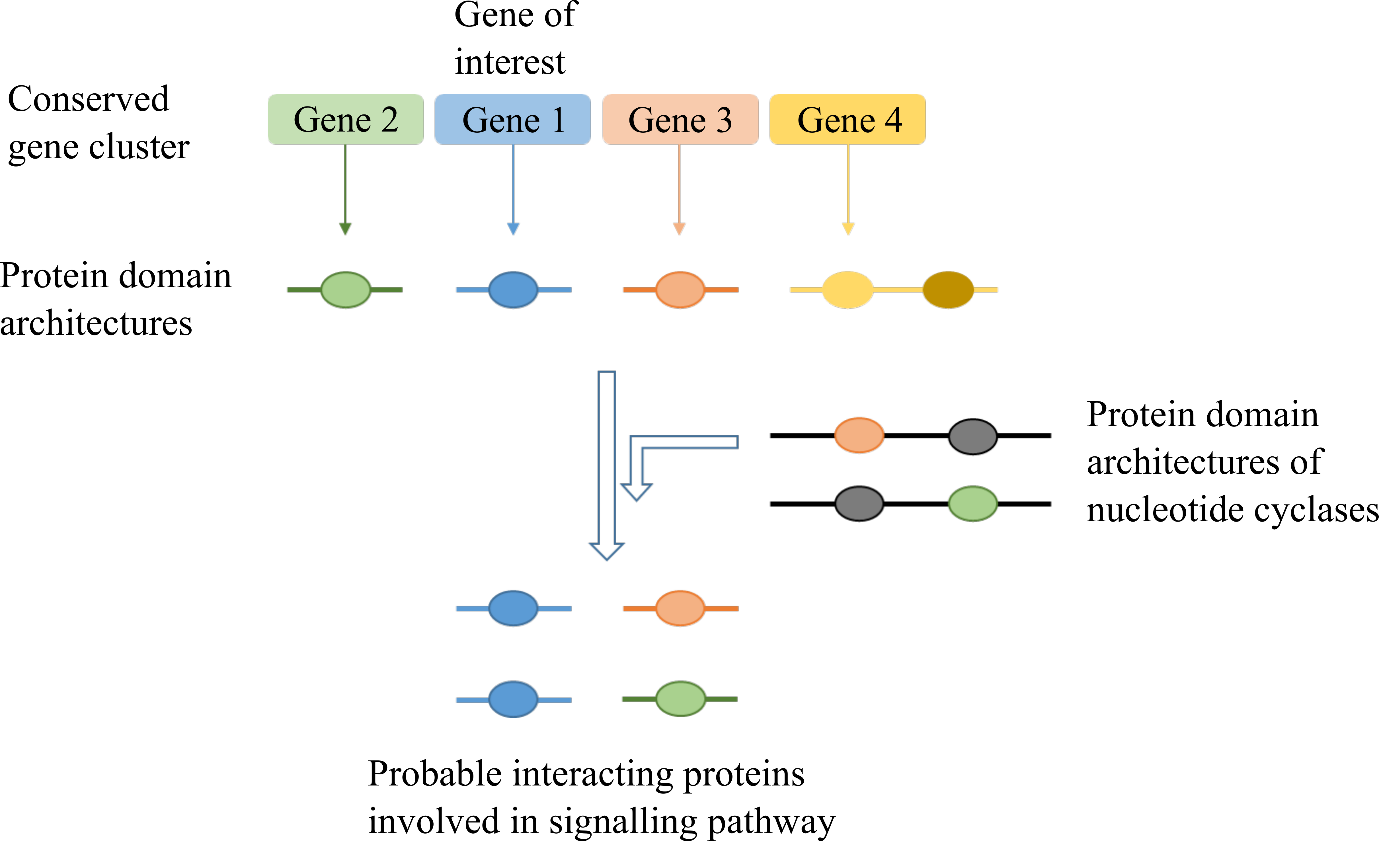


Figure-S4: Inferences from conserved gene neighbourhood. For a gene of interest (blue) that encodes a single domain protein, the protein domain architectures of its evolutionarily conserved gene neighbours, genes 2, 3 and 4, are shown. By means of comparative assessment with the domain organizations in established cyclases (cyclase domain is shown in black) interacting protein partners are predicted. Based on the predicted interacting partners of gene of interest, as illustrated, genes 1 (blue), 2 (green) and 3 (orange) are functionally coupled.

**Supplementary Table**

Table-S1: List of gene records not considered in the analysis due to their questionable completeness

| **UniProt ID** | **Gene name** | **Protein length (aa)** | **Pfam domain family [region]** | **Organism** |
| --- | --- | --- | --- | --- |
| A0A040DE36 | AJ28_02503 | 129 | DUF2652  [1-33] | *M. tuberculosis* MD15956 |
| A0A047RTL8 | V734_02377 | 129 | DUF2652  [1-33] | *M. tuberculosis* OFXR-28 |
| A0A056EVR5 | X094_02653 | 129 | DUF2652  [1-33] | *M. tuberculosis* BTB05-013 |
| A0A056FTF4 | X096_00403 | 129 | DUF2652  [1-33] | *M. tuberculosis* BTB05-348 |
| A5U5R2 | MRA_2591 | 176 | DUF2652  [1-80] | *M. tuberculosis* H37Ra |
| F8M2R3 | MAF_25780 | 98 | DUF2652  [34-98] | *M. africanum* GM041182 |
| F8M2R4 | MAF_25790 | 129 | DUF2652  [1-33] | *M. africanum* GM041182 |
| G0TQ41 | MCAN_26031 | 98 | DUF2652  [34-98] | *M. canettii* CIPT 140010059 |
| G0TQ42 | MCAN_26041 | 129 | DUF2652  [1-33] | *M. canettii* CIPT 140010059 |
| I0AL03 | IALB_1954 | 88 | DUF2652  [33-88] | *Ignavibacterium album* |
| R4M077 | I917_18080 | 97 | DUF2652  [34-76] | *M. tuberculosis* Haarlem/NITR202 |
| R4M8B1 | I917_18085 | 168 | DUF2652  [1-72] | *M. tuberculosis* Haarlem/NITR202 |
